# Impact of Dietary *Lactobacillus plantarum* on Muscle Composition, Hemolymph Biochemistry, Lipid Metabolism, and the mTOR Signaling Pathway in Red Claw Crayfish (*Cherax quadricarinatus*)

**DOI:** 10.3390/ani16060971

**Published:** 2026-03-20

**Authors:** Qin Zhang, Qinghui Zeng, Jiahao Zhao, Luoqing Li, Yijun Hu, Tong Tong, Yongqiang Liu, Dapeng Wang, Rui Wang, Huizan Yang

**Affiliations:** 1 Guangxi Key Laboratory for Polysaccharide Materials and Modifications, Guangxi Marine Microbial Resources Industrialization Engineering Technology Research Center, School of Marine Sciences and Biotechnology, Guangxi Minzu University, 158 University Road, Nanning 530008, China; zhangqin@gxmzu.edu.cn (Q.Z.); zengqinghui@stu.gxmzu.edu.cn (Q.Z.); zhaojiahao@stu.gxmzu.edu.cn (J.Z.); liluoqing@stu.gxmzu.edu.cn (L.L.); huyijun@stu.gxmzu.edu.cn (Y.H.); tongtong@gxmzu.edu.cn (T.T.); 2 Guangxi Key Laboratory for Aquatic Genetic Breeding and Healthy Aquaculture, Guangxi Academy of Fishery Science, 8 Qingshan Road, Nanning 530021, China; raywongxx@163.com (R.W.); yhzyang@163.com (H.Y.)

**Keywords:** probiotic supplementation, hemolymph physiological indices, lipid metabolism regulation, muscle nutritional quality, fatty acid profile, crustacean aquaculture

## Abstract

The objective of this research was to examine how varying dietary inclusion levels of *Lactobacillus plantarum* influence the muscle composition, hemolymph biochemical indices, lipid metabolism, and the mTOR signaling pathway in red claw crayfish (*Cherax quadricarinatus*). Four diets with 0 (CK), 0.10 (LG), 1.00 (MG), and 10.00 (HG) g/kg *L. plantarum* were formulated, with three replicates of 40 crayfish (average weight: 0.13 ± 0.01 g, average length: 0.58 ± 0.01 cm) in a 56-day trial. Results showed that no significant differences in muscle crude protein, crude lipid, ash, or moisture were observed between experimental and CK groups (*p* > 0.05), while the contents of multiple essential amino acids (e.g., arginine up to 6.05%, histidine up to 7.52%) and non-essential amino acids (e.g., aspartic acid up to 3.70%, glutamic acid up to 1.76%) were significantly elevated, and the content of muscle C18:0 (a saturated fatty acid) was notably reduced (*p* < 0.05), while total saturated fatty acids showed no significant variation among all groups (*p* > 0.05). Hemolymph alkaline phosphatase, transaminases, lactate dehydrogenase, and lysozyme activities, as well as glucose, total protein, and albumin levels, were significantly higher in experimental groups (*p* < 0.05). Lipid metabolism was upregulated, and the mTOR pathway was inhibited in experimental crayfish (*p* < 0.05). This study demonstrates that dietary *L. plantarum* enhances lipid metabolism in red claw crayfish, with 1.0 g/kg *L. plantarum* identified as the optimal supplementation level.

## Simple Summary

Red claw crayfish (*Cherax quadricarinatus*) is a valuable freshwater economic crustacean, yet its aquaculture is limited by unstable seedling supply and lipid metabolism disorders, while the regulatory mechanism of *Lactobacillus plantarum* on this species remains unclear. This study conducted a 56-day feeding trial on juvenile crayfish (0.13 ± 0.01 g) with four diets containing 0, 0.10, 1.00 and 10.00 g/kg *L. plantarum*. Results showed *L. plantarum* increased muscle amino acids, reduced saturated fatty acids and improved hemolymph immune and metabolic indices. It also upregulated lipid metabolism and inhibited the mTOR pathway in crayfish. Notably, 1.0 g/kg was the optimal supplementation level.

## 1. Introduction

The red claw crayfish (*Cherax quadricarinatus*) has become a globally significant freshwater economic crustacean due to its large size, rapid growth, high meat yield and nutritional richness, occupying an important position in aquaculture [1,2]. However, the current aquaculture practices for red claw crayfish still face a series of bottlenecks constraining the industry’s sustainable development: at the seedling stage, due to limitations in natural breeding conditions and the still imperfect artificial breeding techniques, the supply of high-quality crayfish seedlings is unstable. Furthermore, they are susceptible to stress during transportation and the initial farming period, resulting in low survival rates [3]. During the rearing phase, the absence of a scientific, standardized feeding system results in inefficient feed conversion, with uneaten feed contaminating water quality. In addition, inadequate nutritional provision may trigger lipid metabolism disorders, subsequently impairing both growth performance and muscle quality in the crayfish. Given these industry challenges, nutritional regulation has become an urgent measure to improve the growth, metabolic status and health of red claw crayfish, and probiotic supplementation is a promising biological regulation strategy for this purpose. *Lactobacillus plantarum* is widely used in aquatic applications as a feed supplement [4,5]. Therefore, we add the widely used *Lactobacillus plantarum* to the feed, aiming to regulate the metabolic and immune-related pathways of the mantis shrimp, thereby effectively improving its nutritional quality and physiological health level.

Probiotics, as promising biological agents in aquaculture, enhance growth performance in aquatic animals by regulating intestinal enzyme activity. This facilitates the absorption of carbohydrates, lipids and other nutrients, thereby providing the organism with more abundant energy [6,7]. *Lactobacillus plantarum*, as a common probiotic, has been demonstrated to enhance feed utilization, regulate gut microbiota and promote growth in various aquatic animals [8,9]. For example, in the pacific white shrimp (*Litopenaeus vannamei*) [10], blunt snout bream (*Megalobrama amblycephala*) [11], and tilapia (*Oreochromis mossambicus*) [12], the addition of *L. plantarum* demonstrated positive effects on digestive metabolism and growth performance. Notably, such beneficial effects of *L. plantarum* are closely linked to its regulatory role in lipid metabolic processes in aquatic hosts, a mechanism that has been validated in multiple species but remains poorly understood in red claw crayfish. However, systematic research remains lacking on the role of *L. plantarum* in red claw crayfish, particularly regarding its regulatory mechanisms on muscle quality, hemolymph biochemical status, and underlying metabolic pathways.

Lipid metabolism is a key physiological process influencing the growth, development and muscle quality of the red claw crayfish, involving not only energy storage but also close correlations to cellular structure and overall health [13,14]. Previous studies have demonstrated a significant correlation between dietary lipid levels and lipid deposition in the pacific white shrimp, whilst the inclusion of probiotics aids in improving lipid metabolism and preventing excessive lipid accumulation [15]. At the molecular mechanism level, the mammalian target of rapamycin (mTOR) signaling pathway, as a key pathway regulating cell growth, metabolism and autophagy, plays a central role in integrating nutritional and energy signals [16]. Recent studies have revealed that certain probiotics can modulate host metabolism and immune responses by influencing the phosphorylation levels of proteins associated with the mTOR pathway. For instance, *Rhodotorula mucilaginosa* enhanced mTOR phosphorylation in pacific white shrimp [17]. *Bacillus coagulans* attenuated phosphorylation within the PI3K/Akt/mTOR pathway in house mouse (*Mus musculus*), thereby inhibiting the phosphorylation of downstream factors [18]. *Bacillus subtilis* activated the ubiquitin-mediated proteolytic pathway, and its inhibition of the mTOR signaling pathway modulated immunity in sea cucumber (*Apostichopus japonicus*) [19]. However, whether probiotics participate in regulating lipid metabolism via the mTOR signaling pathway in red claw crayfish remains unreported and deserves further investigation.

Consequently, the present research is designed to evaluate how dietary supplementation with *L. plantarum* influences the muscle composition, hemolymph biochemical indices, lipid metabolism, and the mTOR signaling pathway in red claw crayfish. By analyzing muscle nutritional composition, hemolymph metabolite changes, key lipid metabolism gene expression, and the expression and regulatory role of mTOR signaling pathway-related molecules at different dietary supplementation levels, we aim to elucidate the underlying mechanisms by which *L. plantarum* improves growth and muscle quality in the red claw crayfish. This is the first integrated analysis of the associations between L. plantarum, lipid metabolism, and the mTOR pathway in red claw crayfish, which enriches the theoretical basis for probiotic-mediated regulation of crustacean nutritional metabolism and provides scientific support for the healthy aquaculture and precision nutritional management of this economic crustacean.

## 2. Materials and Methods

### 2.1. Experimental Diet

The bacterial strain utilized in the current research was *L. plantarum* (No. GXF5101), which was isolated from the intestinal lumen of coho salmon (*Oncorhynchus kisutch*) on account of its rapid growth property and high acidogenic capacity [20].

With reference to the protocol established by Guo et al. [21], *L. plantarum* was cryopreserved in MRS broth (De Man, Rogosa & Sharpe) supplemented with 30% (*v*/*v*) sterile glycerol at −80 °C for long-term maintenance. Primary isolation was carried out by streaking the bacterial culture onto MRS agar plates, which were thereafter incubated anaerobically at 37 °C for 48 h. A single isolated colony was selected randomly, inoculated aseptically into liquid MRS medium, and then cultured aerobically in an orbital shaker incubator (37 °C, 180 rpm) for 24 h to enter the logarithmic growth phase.

Bacterial cells were collected through centrifugation at 4 °C (4000× *g*, 10 min). After discarding the supernatant, the bacterial pellet was rinsed three times with phosphate-buffered saline (PBS), transferred into a 50 mL centrifuge tube, and lyophilized in a vacuum freeze dryer for 48 h to obtain L. plantarum powder with a viable bacterial count of ≥10^10^ CFU/g. The lyophilized powder was preserved at −80 °C until it was used for subsequent experiments.

Four experimental diets were formulated with gradient levels of *L. plantarum* supplementation: control group (CK, 0 g/kg *L. plantarum*), low concentration group (LG, 0.10 g/kg *L. plantarum*), medium concentration group (MG, 1.00 g/kg *L. plantarum*), and high concentration group (HG, 10.00 g/kg *L. plantarum*).

The basic dietary ingredients used in the experiment were provided by Guangdong Hengxing Feed Industry Co., Ltd. (Guangzhou, China). The ingredients were crushed, sieved through a 60-mesh screen, and thoroughly mixed with lyophilized *L. plantarum* powder at the concentrations prior to granulation. The diets were vacuum-dried to maintain moisture content below 10%, then stored in the dark at −20 °C, and freshly prepared every 4 days to ensure physicochemical stability and prevent microbial contamination, with the viability of live *L. plantarum* in the stored feed quantified weekly via plate counting to verify probiotic activity.

Four dietary treatments were prepared: a control group (CK) without *L. plantarum*, and three supplemented groups containing 0.10, 1.00, and 10.00 g/kg of *L. plantarum*, designated as LG, MG, and HG, respectively. These diets were preserved in sealed bags at −20 °C, and new batches were prepared on a weekly basis. To ensure bacterial stability during storage, the viability of live *Lactobacillus plantarum* in the feed was assessed weekly by plate counting. Detailed nutritional compositions are presented in Table 1.

### 2.2. Animal Farming and Sampling

The red claw crayfish used in the experiment were sourced from the South Breeding Base of the Guangxi Academy of Fisheries Sciences (Nanning, China). The experimental protocol of the present study was endorsed by the Biomedical Ethics Committee of Guangxi Minzu University (Nanning, China) with the approval number GXMZU-2023-018.

Prior to the experiment, potassium permanganate solution at a concentration of 10 mg/L was used for disinfecting the interior and exterior of the rearing tanks, as well as the recirculating water system. Before the formal experiment, all juvenile red claw crayfish were acclimated for 7 days in the recirculating water system under natural light conditions. The environmental parameters were controlled as follows: water temperature 26.5 ± 0.5 °C, dissolved oxygen content > 6 mg/L, ammonia nitrogen content < 0.5 mg/L, and pH 7.5–8.0.

After acclimation, healthy juvenile crayfish with uniform body size, no limb damage, and active behavior were selected for the formal experimental phase. This experiment employed a completely randomized design, encompassing four distinct treatment groups: control group (CK, 0 g/kg *L. plantarum*), low concentration group (LG, 0.10 g/kg *L. plantarum*), medium concentration group (MG, 1.00 g/kg *L. plantarum*), and high concentration group (HG, 10.00 g/kg *L. plantarum*). Each treatment group consisted of three replicates, utilizing a total of 12 independent aquaculture cages (sized 2 m × 4 m × 2 m), with the water depth within the cages maintained at 0.7 m. Each cage was stocked with 50 red claw crayfish of (average weight: 0.13 ± 0.01 g, average length: 0.58 ± 0.01 cm). In total, 600 juvenile crayfish were stocked in the 12 cages.

During the formal experimental phase, the environmental conditions of the aquaculture water body were maintained consistent with those during the acclimation phase. The daily ration was maintained at approximately 5% of the crayfish body weight and adjusted as necessary based on actual feed intake. Before each feeding event, the growth status and any mortality of crayfish in each tank were systematically recorded. Following the morning feeding, one-third of the tank water was replenished, during which fecal matter and uneaten feed residues were removed. The feeding trial was conducted over a 56-day period.

At the end of the 56-day feeding trial, the survival rate of crayfish in all groups was above 89% and the survival rates and final weight of crayfish are shown in Section 3.1. No significant cannibalistic behavior was observed in any of the experimental groups throughout the trial period; the low risk of cannibalism was attributed to three key factors: (1) the provision of sufficient daily feed (5% of body weight, adjusted according to actual intake) to eliminate food competition, (2) strict maintenance of stable water quality parameters (e.g., dissolved oxygen > 6 mg/L, low ammonia nitrogen) to reduce stress-induced aggressive behavior, and (3) the selection of uniformly sized juvenile crayfish with no physical damage, which minimized inter-individual competitive and aggressive interactions. At 56 days rearing period, all red claw crayfish were subjected to a 24 h fasting period. First, an anticoagulant solution was prepared with glucose (20.5 g/L), sodium citrate (8 g/L), and sodium chloride (4.2 g/L), adjusted to pH 7.5, and then pre-chilled for later use. From each replicate, nine red claw crayfish were selected, rinsed with sterile physiological saline, and placed on ice for 15 min to induce anesthesia. Hemolymph was extracted from the base of the fifth abdominal appendage and stored in sterile, enzyme-free 1.5 mL Eppendorf tubes. Centrifugation was performed at 3500 g and 4 °C for 10 min. The supernatant hemolymph was collected and stored at −80 °C for subsequent analysis. Subsequently, ice-bathed dissection was performed to harvest muscle and hepatopancreas tissues, which were rapidly frozen in liquid nitrogen. All collected samples were stored at −80 °C for subsequent analysis.

### 2.3. Analysis of Muscle Composition

Abdominal muscle tissue from three crayfish per tank was pooled as test samples for the determination of conventional muscle composition. The crude protein, crude lipid, moisture and ash contents in the muscle of red claw crayfish was analyzed following the standard methodologies of the Association of Official Analytical Chemists [22]. In simple terms, the crude protein content was determined using the micro-Kjeldahl method, the crude lipid content was determined using the Soxhlet extraction method, the moisture content was determined by oven-drying at 105 °C, and the ash content was determined using the high-temperature incineration method.

Quantification of total and free fatty acid levels in the muscle was performed in accordance with the China National Standard GB 5009.168-2016 for determining fatty acids in food products [23]. The fatty acid composition of muscle tissue was analyzed using gas chromatography with flame ionization detection (GC-FID). For this analysis, lipids were first extracted using a chloroform-methanol mixture (2:1, *v*/*v*). The extracted lipids were then subjected to methylation with boron trifluoride in methanol (BF_3_-MeOH) to produce fatty acid methyl esters (FAMEs). Furthermore, the hydrolyzed amino acid profile, comprising 17 different analytes, was assessed. This analysis was carried out on a Hitachi L-8900 amino acid analyzer following acid hydrolysis with 6 mol/L HCl at 110 °C for 24 h, adhering to the China National Standard GB 5009.124-2016 for amino acid determination in foods [24].

### 2.4. Analysis of Hemolymph Physiological and Biochemical Parameters

Three samples of blood lymph supernatant that had been centrifuged and stored at minus 80 degrees Celsius were thawed and used for analysis. The hemolymph physiological and biochemical parameters of red claw crayfish were analyzed following the method described by Liu et al. [25] Measurements were conducted on a Rayto RT-6100 ELISA analyzer (Shenzhen, China), using commercial reagent kits from Nanjing Jiancheng Bioengineering Institute (Nanjing, China). Lysozyme (LZM) concentration in the hemolymph was determined through a turbidimetric assay, with results expressed in μg/mL. Alkaline phosphatase (AKP) activity was quantified by microplate enzymatic methods. AKP activity was expressed in King units per 100 mL, defined as the enzyme quantity generating 1 mg of phenol per 100 mL of hemolymph after a 15 min substrate reaction at 37 °C (King unit/100 mL). The aspartate aminotransferase (AST) and alanine aminotransferase (ALT) activities were quantified using a microplate technique, with results expressed in U/L. Lactate dehydrogenase (LDH) activity was also determined on a microplate, and one unit corresponds to the generation of 1 μmol of pyruvate per liter of hemolymph following a 15 min incubation with the substrate at 37 °C (U/L). For total protein (TP) assessment, the bicinchoninic acid microplate assay was employed, and concentrations are given in g/L. Albumin (ALB) concentration was analyzed by an enzymatic microplate method (g/L). Glucose (GLU) levels were determined by the glucose oxidase procedure (mmol/L). Triglycerides (TG) were determined by the glycerol-3-phosphate oxidase-phenol amino phenazone enzymatic method (mmol/L). Total cholesterol (T-CHO) was measured via the cholesterol oxidase-phenol amino phenazone method (mmol/L). Comprehensive methodological details are available at http://www.njjcbio.com/ (accessed on 8 January 2026).

### 2.5. Analysis of Relative Expression Levels of Genes

In accordance with the method described by Zhang et al. [26], real-time quantitative polymerase chain reaction (RT-qPCR) was utilized to assess target gene expression levels under RNase-free conditions. Quantitative RT-qPCR analysis was performed to measure hepatopancreas mRNA expression of rapamycin target protein 1 (*mtor1*), rapamycin target protein 2 (*mtor2*), protein kinase B (*akt*), acetyl-CoA carboxylase (*acc*), carnitine palmitoyl transferase 1 (*cpt1*), adiponectin receptor (*adipor*), adenosine monophosphate-activated protein kinase (*ampk*) α/β/γ subunits, peroxisome proliferator-activated receptor γ (*pparγ*), sterol regulatory element-binding protein (*srebp*), and fatty acid synthase (*fas*) genes. *β-actin* served as the non-regulated reference gene. Gene-specific primers were designed using Primer Premier 6.0 software, based on mRNA sequences of the red claw crayfish obtained from the National Center for Biotechnology Information (NCBI) database, and subsequently synthesized by Shanghai Sangon Biotech Co., Ltd. in Shanghai, China. The primer sequences for RT-qPCR are presented in Table 2.

The experimental procedure was performed as follows: Firstly, total RNA extraction: Total RNA was isolated from hepatopancreas tissues (approximately 100 μg) using the Takara MiniBEST Universal RNA Extraction Kit (Takara Bio Inc., Shiga, Japan, Dalian, China), following the manufacturer’s protocol. RNA concentration and purity were subsequently assessed with a ND-2000 spectrophotometer (Thermo Fisher Scientific, Waltham, MA, USA). The A260/A280 absorbance ratio served as the primary metric for evaluating RNA purity, where a value between 1.8 and 2.0 was indicative of high-purity RNA. Secondly, for the reverse transcription (RT) reaction, 1 μg of high-quality total RNA was converted into complementary DNA (cDNA) using the PrimeScript™ RT Reagent Kit (Perfect Real Time; Takara Bio Inc., Dalian, China). The RT procedure was carried out under the following thermal cycling conditions: primer annealing at 30 °C for 10 min, cDNA synthesis at 60 °C for 30 min, enzyme inactivation at 95 °C for 5 min, and a final hold at 5 °C for 5 min, in a single cycle. Finally, RT-qPCR analysis: RT-qPCR was performed using a LightCycler 96 system (Roche Diagnostics, Basel, Switzerland). Each 20 μL reaction mixture contained 10 μL of SYBR Premix Ex Taq (Takara Bio Inc., Dalian, China), 0.5 μL each of forward and reverse primers, 1 μL of cDNA template, and 8 μL of double-distilled water (ddH_2_O). The thermal cycling protocol comprised an initial denaturation at 95 °C for 10 s, followed by 40 cycles of denaturation at 95 °C for 60 s, annealing at 60 °C for 30 s, and extension at 72 °C for 90 s. A melting curve analysis was conducted at 95 °C post-amplification to confirm PCR product specificity.

The 2^−∆∆Ct^ method [27] was applied to calculate the relative expression levels of *mtor1*, *mtor2*, *akt*, *acc*, *cpt1*, *adipor*, *ampkα*, *ampkβ*, *ampkγ*, *pparγ*, *srebp*, and *fas* genes in the hepatopancreas of red claw crayfish.

### 2.6. Data Calculation and Statistics

All experimental datasets were initially curated using Microsoft Excel (Microsoft 365, Microsoft Corporation, Redmond, WA, USA). Prior to statistical evaluation, the Shapiro–Wilk test was used to assess the normality of data distribution, and the Levene’s test was performed to verify the homogeneity of variance, so as to confirm compliance with the assumptions for parametric tests. Subsequent analyses were performed with IBM SPSS Statistics 27.0 (International Business Machines Corporation, Armonk, NY, USA). Differences among multiple groups were analyzed by one-way analysis of variance (ANOVA), with post hoc comparisons conducted using Tukey’s multiple range test. For graphical representation of data, the least significant difference (LSD) method was employed to determine statistical significance. A two-tailed *p*-value of less than 0.05 was considered statistically significant. Data are presented as mean ± standard deviation (mean ± SD).

## 3. Result

### 3.1. Effects of Lactobacillus plantarum Supplementation on Survival Rate and Final Weight in Red Claw Crayfish

Compared with the control group (CK group), the survival rate and final weight of red claw crayfish in the LG, MG, and HG groups were significantly higher than those in the CK group (*p* < 0.05), as shown in Table 3.

### 3.2. Effects of Lactobacillus plantarum Supplementation on Muscle Composition and Nutritional Components in Red Claw Crayfish

Compared with the control group (CK group), there were no significant differences in the crude protein, crude lipid, ash, or moisture contents among the MG, LG, and HG groups (*p* > 0.05), as shown in Table 4.

Among the essential amino acids, the arginine, histidine, leucine, lysine, methionine, and valine contents were significantly higher in all treatment groups relative to the CK group (*p* < 0.05). Isoleucine and phenylalanine contents were significantly higher in the MG and HG groups than in the CK group (*p* < 0.05), but no significant difference was observed between the LG and CK groups (*p* > 0.05). In contrast, no significant differences were found in threonine, and total essential amino acid content across all groups (*p* > 0.05), as shown in Table 5.

Among non-essential amino acids, aspartic acid, glutamic acid, and total non-essential amino acids contents were significantly higher in all treatment groups relative to the CK group (*p* < 0.05). Alanine and Serine contents were significantly higher in the MG and HG groups than in the CK group (*p* < 0.05), but no significant difference was observed between the LG and CK groups (*p* > 0.05). Proline content was significantly higher in the LG and HG groups than in the CK group (*p* < 0.05), but no significant difference was observed between the MG and CK groups (*p* > 0.05). In contrast, no significant differences were found in glycine and tyrosine across all groups (*p* > 0.05), as shown in Table 5.

Among the saturated fatty acids, the C18:0 content in the HG group was significantly lower than that in the CK group (*p* < 0.05), while no significant differences were observed among the LG, MG and CK groups (*p* > 0.05). No significant variations were detected among all groups in the contents of C14:0, C16:0, C17:0, and total saturated fatty acids (∑SFAs) (*p* > 0.05), as shown in Table 6.

Among the monounsaturated fatty acids, the contents of C16:1n-7, C18:1n-9, and total monounsaturated fatty acids (∑MUFAs) were significantly higher in all treatment groups relative to the CK group (*p* < 0.05), as shown in Table 6.

Among the polyunsaturated fatty acids, the contents of C20:2n-9, C18:3n-6, and C20:3n-6 in the HG and MG groups were significantly higher than those in the CK group, while no significant differences were observed between the LG and CK groups (*p* > 0.05). The contents of C18:2n-6, arachidonic acid (ARA, C20:4n-6), C22:4n-6, total n-6 polyunsaturated fatty acids (∑n-6 PUFAs), C18:3n-3, eicosapentaenoic acid (EPA, C20:5n-3), C22:5n-3, docosahexaenoic acid (DHA, C22:6n-3), total n-3 polyunsaturated fatty acids (∑n-3 PUFAs), total polyunsaturated fatty acids (∑PUFAs), total long-chain polyunsaturated fatty acids (∑LC-PUFAs), n-3/n-6 ratio, DHA + EPA, and polyunsaturated fatty acids (including EPA and DHA) were significantly higher in all treatment groups relative to the CK group (*p* < 0.05), as shown in Table 6.

### 3.3. Effects of Lactobacillus plantarum Supplementation on Biochemical Parameters of Hemolymph in Red Claw Crayfish

Compared with the CK group, the contents (activities) of AKP, TP, and LZM in the LG, MG, and HG groups were significantly higher than those in the CK group (*p* < 0.05). The contents of GLU and ALB in the MG group were significantly higher than those in the CK group (*p* < 0.05), while no significant differences were observed among the LG, HG and CK groups (*p* > 0.05). The activities of AST, ALT, and LDH in the LG, MG, and HG groups were significantly lower than those in the CK group (*p* < 0.05). There were no significant differences in the contents (activities) of CHE and T-CHO among the MG, LG, HG, and CK groups (*p* > 0.05), as shown in Table 7.

### 3.4. Effects of Lactobacillus plantarum Supplementation on Lipid Metabolism-Related Gene Expression in Red Claw Crayfish

**Figure 1:**
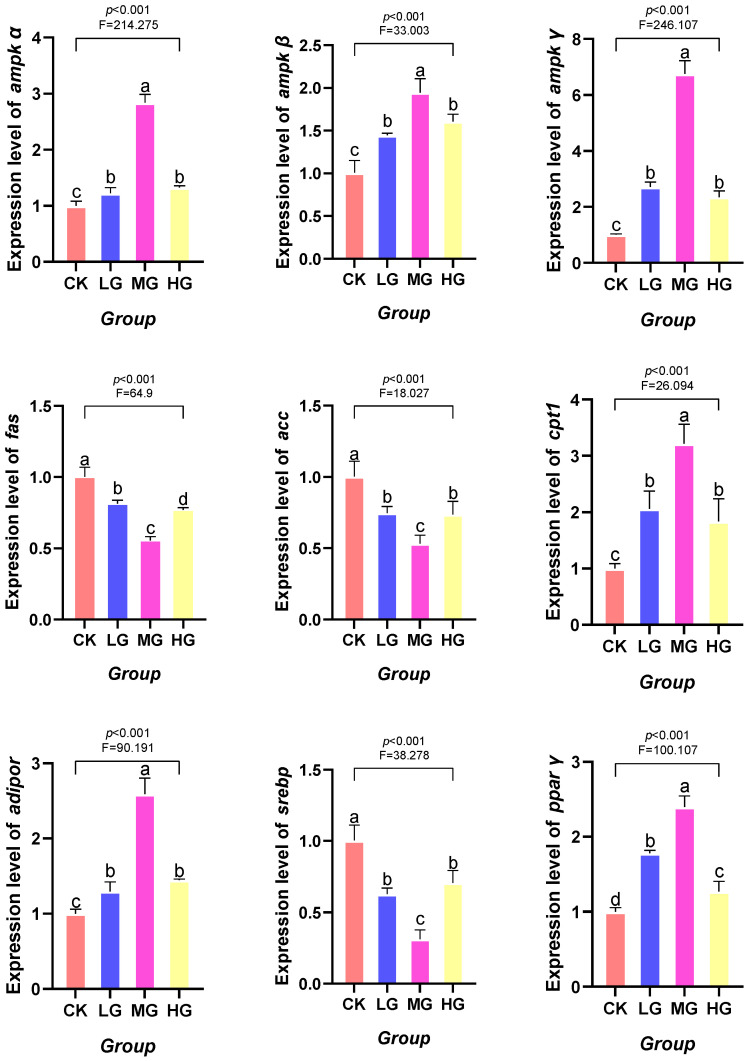
Effect of *Lactobacillus plantarum* supplementation in feed on the mRNA expression levels of fatty acid synthase (*fas*), acetyl-CoA carboxylase (*acc*), carnitine palmitoyl transferase 1 (*cpt1*), adenosine monophosphate-activated protein kinase alpha (*ampkα*), *ampkβ*, *ampkγ*, peroxisome proliferator-activated receptor γ (*pparγ*), adipokine receptor (*adipor*), and sterol regulatory element-binding protein (*srebp*) genes in the hepatopancreas of red claw crayfish. All data represent mean ± standard deviation (*n* = 3). Different superscript letters in the figure indicate significant differences among the data (*p* < 0.05).

As the dietary supplementation level of *L. plantarum* increased, the mRNA expression levels of *ampkα*, *ampkβ*, *ampkγ*, *pparγ*, *adipor*, and *cpt1* genes displayed a trend of first increasing and then decreasing. In contrast, the mRNA expression levels of *fas*, *acc*, and *srebp* genes exhibited an opposite trend: first decreasing and then increasing. Specifically, the mRNA expression levels of *ampkα*, *ampkβ*, *ampkγ*, *pparγ*, *adipor*, and *cpt1* genes in the LG, MG, and HG groups were significantly higher than those in the CK groups (*p* < 0.05). Conversely, the mRNA expression levels of *fas*, *acc*, and *srebp* genes in the LG, MG, and HG groups were significantly lower than those in the CK group (*p* < 0.05), as shown in Figure 1.

### 3.5. Effects of Lactobacillus plantarum Supplementation on AKT/mTOR Signaling Pathway-Related Gene Expression in Red Claw Crayfish

**Figure 2:**
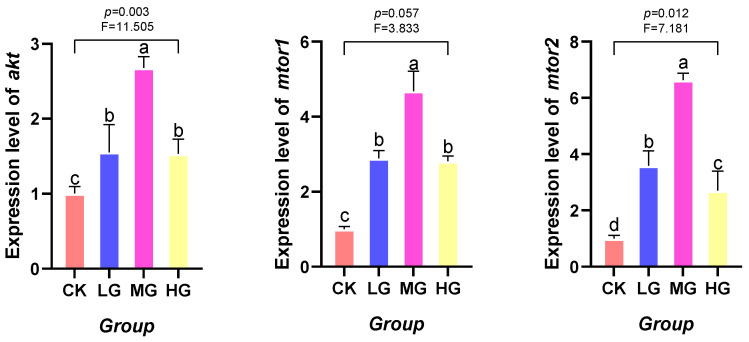
Effects of *Lactobacillus plantarum* supplementation in feed on the mRNA expression levels of mammalian target of rapamycin 1 (*mtor1*), mammalian target of rapamycin 2 (*mtor2*), and protein kinase B (*akt*) in the hepatopancreas of red claw crayfish. All data represent mean ± standard deviation (*n* = 3). Different superscript letters in the figure indicate significant differences among the data (*p* < 0.05).

As the dietary supplementation level of *L. plantarum* increased, the mRNA expression levels of *akt*, *mtor1*, and *mtor2* genes exhibited a trend of initial decrease followed by increase. Specifically, the mRNA expression level of *akt* gene in the LG, MG, and HG groups was significantly lower than that in the CK groups (*p* < 0.05). The mRNA expression levels of *mtor1* and *mtor2* genes in the MG group were significantly lower than those in the CK groups (*p* < 0.05), while no significant differences were observed among the LG, HG and CK groups (*p* > 0.05), as shown in Figure 2.

## 4. Discussion

The muscular composition of the red claw crayfish significantly influences its edible quality and nutritional profile, while alterations in feed formulation are known to impact the nutrient composition of muscle tissue [28]. The present study demonstrated that dietary supplementation with *L. plantarum* did not markedly alter the muscle composition of red claw crayfish. No statistically significant differences were observed between the experimental and control groups in terms of muscle crude protein, crude lipid, ash, or moisture content. This stability in muscle constituents may be attributed to the inherent homeostatic regulation of protein and lipid levels within the crayfish’s muscle tissue. Furthermore, these constituents, which are intrinsically linked to muscle tissue microstructure, are relatively resistant to short-term modulation by dietary additives [29,30]. As a probiotic, *L. plantarum* primarily exerts its effects within the intestinal tract, indirectly enhancing growth performance by improving feed utilization and promoting gastrointestinal health [31], rather than directly engaging in muscular or mineral synthesis and deposition. These observations aligned with reports from other aquatic species, such as sobaity bream (*Sparidentex hasta*) and greater amberjack (*Seriola dumerili*), in which *L. plantarum* supplementation similarly failed to induce significant changes in muscle composition [32,33]. However, several studies reported that *L. plantarum* can modulate ash and moisture levels in fish species, including turbot (*Scophthalmus maximus* L.) and bighead catfish (*Clarias macrocephalus*) [34,35]. These discrepancies may arise from factors such as bacterial strain specificity, host organism differences, dosage, microbial metabolic activity, and experimental conditions.

This study demonstrated that the inclusion of *L. plantarum* in feed significantly enhanced the content of both essential and non-essential amino acids in the muscle tissue of red claw crayfish. Amino acids serve as the fundamental building blocks for protein synthesis, and their composition and concentration directly determine the nutritional value of muscle tissue [36]. The endogenous digestive enzyme systems of aquatic animals are often insufficient to fully break down feed proteins. Exogenous probiotics can utilize their proteolytic activity to degrade macromolecular proteins in feed into smaller peptides and free amino acids that are more readily absorbed, thereby enhancing amino acid utilization [37]. The findings of this study aligned with those of several previous investigations, such as those conducted on largemouth bass (*Micropterus salmoides*) [38] and white shrimp (*Litopenaeus vannamei*) [39], in which the addition of probiotics significantly elevated muscle amino acid levels, thereby improving the amino acid composition and nutritional quality of the muscle tissue.

Concurrently, the addition of *Lactobacillus plantarum* significantly optimized the fatty acid composition of red claw crayfish muscle. Levels of polyunsaturated fatty acids (PUFAs), monounsaturated fatty acids (MUFAs), and long-chain polyunsaturated fatty acids (LC-PUFAs, such as ARA, EPA, and DHA) in the muscle were all markedly elevated, while saturated fatty acid (SFA) contents remained unchanged. Of particular note, the contents of n-3 PUFAs (such as EPA and DHA) and the n-3/n-6 ratio were significantly elevated. This ratio holds significant importance for anti-inflammatory effects and human cardiovascular health [40]. The increase in MUFAs (such as palmitic acid and oleic acid) aligned with the upregulation of lipid metabolism genes observed in this study, suggesting that *L. plantarum* may promote the deposition of beneficial fatty acids by regulating host lipid metabolic pathways. The findings of this study are consistent with those of the research on Nile tilapia (*Oreochromis niloticus*) [41], where the addition of probiotics significantly increased the fatty acid content in the muscle. This optimization of fatty acid composition not only enhanced the physiological health and stress resistance of the red claw crayfish itself, but also significantly elevated the nutritional value of its muscle as a human food source [42,43].

This study demonstrated that the inclusion of *L. plantarum* in feed significantly elevated glucose levels in the hemolymph of red claw crayfish. As the primary energy source, this increase in glucose concentration may be attributed to *L. plantarum*’s promotion of carbohydrate fermentation and conversion, thereby providing enhanced energy support for growth and protein synthesis [44]. The increase in glucose content aided in maintaining fluid balance and enhancing metabolic efficiency, consistent with previous findings that *L. plantarum* improves glucose metabolism in white shrimp [45] and nile tilapia (*Oreochromis niloticus*) [46].

Regarding indicators reflecting hepatopancreas function, the addition of *L. plantarum* significantly reduced the activity of AST, ALT, and LDH. These enzymes are typically released into the bloodstream when hepatocytes are damaged, and their decreased activity suggests that *L. plantarum* possesses certain hepatoprotective effects [47]. This protective effect may stem from the probiotics’ antioxidant properties, which can mitigate oxidative stress damage to hepatocytes [48,49]. Furthermore, *L. plantarum* or its metabolites may influence LDH activity through mechanisms such as competitive inhibition [50]. It is noteworthy that total cholesterol in hemolymph did not undergo significant alteration in this experiment, which contrasts with reports of cholesterol reduction or elevation in certain studies [51,52]. This clearly indicates that the observed improvement in lipid metabolism in this study was mainly achieved through regulation of triglyceride metabolism and fatty acid utilization, rather than through cholesterol metabolism. This discrepancy may be attributed to species variations, dietary composition, or strain specificity, with the underlying mechanisms warranting further investigation. 

Albumin is a crucial circulating protein in the hemolymph, and its concentration within the hemolymph plays a vital role in the immune response [53,54]. This study indicated that total protein and albumin levels exhibited a trend of initially rising before subsequently declining. The initial increase may be associated with *L. plantarum* promoting intestinal microbial fermentation and generating metabolites that stimulate protein synthesis [55,56]. As nutrient sources are depleted, the growth rate of *L. plantarum* slows, resulting in reduced production of its metabolites. This consequently diminishes its stimulatory effect on total protein and albumin synthesis [57,58]. Similar dynamic changes have been reported in other crustaceans and fish species. For instance, research by Shi et al. [47] demonstrated that supplementing feed with *L. plantarum* significantly increased albumin content in the hemolymph of red swamp crayfish (*Procambarus clarkii*). Pradhan et al. [59] observed a significant increase in both total protein and albumin levels in the hemolymph of the roho labeo (*Labeo rohita*) when brewer’s yeast was incorporated into their diet. This suggests that the effects of *L. plantarum* on protein metabolism may be time dependent. As a crucial osmoregulatory protein and immune-related factor, variations in albumin levels also reflect adjustments in the organism’s nutritional and immune status [60].

Regarding non-specific immune markers, both AKP and LZM activities exhibited a pattern of initial elevation followed by subsequent decline. The initial increase in activity may be attributed to *L. plantarum* acting as an immune activator, providing a short-term stimulation of the body’s immune response [61,62]. As the microbial community stabilizes in later stages, the stimulating effect correspondingly diminishes [63]. This dynamic pattern is consistent with observations from other studies employing probiotics to enhance the immune function of aquaculture animals. For instance, Soltani et al. [64] reported that immunized rainbow trout (*Oncorhynchus mykiss*) fed with *L. plantarum* supplementation exhibited significantly higher AKP and LZM activities compared to the control group receiving basal diet. Wei et al. [5] demonstrated that the addition of *L. plantarum* to Chinese shrimp (*Penaeus orientalis*) farming also exhibited a trend of AKP activity first increasing and then decreasing, with significant differences observed between groups. Pandey et al. [65] found that supplementing the diet of growing common carp (*Cyprinus carpio*) with *L. plantarum* improved fish growth and health, particularly through a significant increase in LZM activity. Furthermore, there were no significant differences in ChE activity among the groups, indicating that *L. plantarum* did not exert a discernible effect on neurotransmitter metabolism under the experimental conditions employed [66]. Analysis indicated that the dosage of *L. plantarum* added during the trial may not have reached a level capable of significantly affecting cholinesterase content [67]. This finding is also consistent with results from certain fish studies, such as that by Singh et al. [68], where the addition of probiotics to the diet did not significantly alter cholinesterase activity in the hemolymph of the roho labeo (*Labeo rohita*).

This study demonstrated that supplementation of feed with *L. plantarum* significantly modulated the expression of key genes involved in lipid metabolism in the red claw crayfish. Specifically, the AMPK pathway was markedly activated, with the expression levels of its α, β, and γ subunit genes all significantly increased. As a central sensor in cellular energy metabolism, AMPK activation inhibits hepatic lipogenesis and promotes fatty acid oxidation [69]. Concurrently, the expression levels of the key transcription factor *srebp* and its downstream target genes *fas* and *acc* were significantly downregulated [70]. This aligned with findings from multiple studies, such as those showing that *L. plantarum* alleviates lipid accumulation in fish hepatopancreass induced by high-carbohydrate diets through AMPK activation, and that it can downregulate *fas* gene expression in largemouth bass [71] and *acc* gene expression in common carp [65]. Therefore, the findings of this study suggested that *L. plantarum* may reduce hepatic lipid accumulation in the red claw crayfish by activating the AMPK pathway, thereby inhibiting the SREBP-dependent lipogenic program.

In promoting lipolysis, this study observed significant alterations in gene expression closely associated with fatty acid β-oxidation. The expression levels of *pparγ* and *cpt1* were markedly upregulated. PPARγ serves as a crucial nuclear transcription factor regulating lipid metabolism [72], CPT-1 is the rate-limiting enzyme responsible for transporting long-chain fatty acids into mitochondria for β-oxidation [73]. The synergistic upregulation of both indicated that *L. plantarum* enhances the fatty acid degradation capacity of the red claw crayfish. This finding resonated with studies in other aquatic species, such as those where *Bacillus subtilis* alleviating lipid metabolism abnormalities in hybrid grouper (*Epinephelus fuscoguttatus* ♀ × *E. lanceolatus* ♂) by upregulating *cpt1* levels, and those demonstrating that activation of PPARα/γ being demonstrated to induce *cpt1* expression for regulating lipid breakdown [74]. Moreover, the expression of *adipor*, which is closely associated with glucose and lipid metabolism, was also significantly upregulated. This further supported the role of *Lactobacillus plantarum* in promoting fatty acid oxidation metabolism [75]. It is consistent with findings from *Coilia nasus* studies indicating that *L. plantarum* upregulates *adipor* to ameliorate lipid dysregulation [76].

This investigation revealed that dietary supplementation with *L. plantarum* markedly suppressed the expression of the AKT and mTOR signaling pathways in red claw crayfish. AKT, a critical serine/threonine kinase, is central to the regulation of growth, metabolism, cell survival, and apoptosis. It additionally modulates immune cell differentiation, autophagy, and energy metabolism through the mTOR complex (mTORC1/2) [77,78]. The observed downregulation of AKT/mTOR pathway-related gene expression suggests a potential link to enhanced autophagic activity, modulated energy utilization, and improved overall physiological status in red claw crayfish, as these pathways are well-documented to mediate such biological processes in crustaceans and other aquatic invertebrates. This result was consistent with prior studies, including those demonstrating that *Bacillus* suppresses the mTOR pathway in sea cucumber (*Apostichopus japonicus*) to potentiate immunomodulation [79], and those demonstrating that *L. plantarum* reduces energy expenditure through inhibition of mTOR signaling [80]. Based solely on the gene expression data obtained in this study, we tentatively propose that *L. plantarum* induced AMPK activation and concurrent mTOR inhibition likely redirected energy away from growth-related anabolic processes toward immune function and lipid metabolism regulation. This energy reallocation provides a cohesive link between the improved hemolymph health markers (elevated AKP, LZM, TP, ALB; reduced AST/ALT) and the molecular changes in the AMPK/mTOR axis: by inhibiting mTOR, the crayfish conserves energy that would otherwise be allocated to rapid growth, and redirects it to support immune competence and efficient lipid metabolism. Such a trade-off is particularly beneficial in aquaculture settings, where balanced immune function and metabolic health are critical for sustaining growth performance and survival, even if the rate of somatic growth is moderately adjusted.

Notably, this study has several limitations that should be acknowledged. First, the molecular mechanisms underlying the reciprocal regulation of AMPK and mTOR by L. plantarum were only explored at the transcriptional level; future studies should investigate protein phosphorylation levels to validate post-translational regulation, which is critical for the functional activation of these signaling pathways. Second, research should aim to delineate the cross-regulatory network linking this pathway to lipid metabolism and immune markers, thereby providing a comprehensive understanding of the mechanistic basis of L. plantarum action. Third, the present study focused on the hepatopancreas as the primary target tissue for metabolic regulation; future research should extend to intestinal and muscle tissues to comprehensively understand the tissue-specific regulatory effects of L. plantarum on lipid metabolism and signaling pathways. Finally, the potential role of intestinal microbiota and their metabolites (e.g., short-chain fatty acids) in mediating the crosstalk between L. plantarum supplementation and the mTOR axis was not explored, which warrants further investigation to clarify the holistic regulatory network.

## 5. Conclusions

In summary, this study demonstrated that supplementing the basal diet with 1 g/kg *L. plantarum* can effectively modulate the muscle amino acid and fatty acid profiles of red claw crayfish. Meanwhile, it exerts regulatory effects on hemolymph glucose metabolism, protein metabolism, and lipid metabolism. Furthermore, this dietary supplementation downregulates the expression of genes associated with the AKT/mTOR signaling pathway, thereby inhibiting the activation of this pathway and subsequently regulating the autophagic process in red claw crayfish. This research preliminarily explores the probiotic functions of *L. plantarum* in red claw crayfish, providing a theoretical basis for the application of probiotics in crayfish aquaculture.

## Figures and Tables

**Table 1:** The approximate nutritional composition of the diets for red claw crayfish (g/kg of dried diet).

Ingredients	Groups			
	CK	LG	MG	HG
*Lactobacillus plantarum*	0	0.10	1.00	10.00
Fish meal	485.00	485.00	485.00	485.00
Soybean meal	119.30	119.20	118.30	109.30
Sorghum flour	106.50	106.50	106.50	106.50
Wheat flour	139.00	139.00	139.00	139.00
Corn flour	45.00	45.00	45.00	45.00
Soy Lecithin	10.00	10.00	10.00	10.00
Fish oil	15.20	15.20	15.20	15.20
Gelatin	20.00	20.00	20.00	20.00
Calcium carbonate	10.00	10.00	10.00	10.00
Choline chloride	5.00	5.00	5.00	5.00
Mineral premixes ^a^	20.00	20.00	20.00	20.00
Vitamin premixes ^b^	20.00	20.00	20.00	20.00
Vitamin C	5.00	5.00	5.00	5.00
Proximal composition (% of diet dry Matter)				
Dry material	91.18 ± 0.80	92.06 ± 0.55	91.33 ± 0.87	92.11 ± 0.49
Ash	12.97 ± 1.33	12.65 ± 0.89	13.36 ± 1.18	12.83 ± 1.20
Ethereal extract	7.47 ± 0.60	8.10 ± 0.35	7.56 ± 0.78	7.89 ± 0.58
Crude protein	39.09 ± 1.56	38.23 ± 2.09	38.57 ± 1.49	39.18 ± 1.89
Crude lipid	7.88 ± 0.66	7.84 ± 0.35	7.57 ± 0.44	7.75 ± 0.86
Fiber	1.27 ± 0.15	1.31 ± 0.26	1.28 ± 0.21	1.25 ± 0.18

**Note:** ^a^ Mineral premixes (mg/kg): KCl, 0.5; MgSO_4_·7H_2_O, 0.5; ZnSO_4_·7H_2_O, 0.09; MnCl_2_·4H_2_O, 0.0234; CuSO_4_·5H_2_O, 0.005; KI, 0.005; CoCl_2_·2H_2_O, 0.0025; Na_2_HPO_4_, 2.37. ^b^ Vitamin premixes (mg/kg): Vitamin B_12_, 0.02; Vitamin A acetate, 5000 IU; Vitamin D_3_, 4000 IU; α-tocopherol acetate, 100 IU; menadione, 5; thiamine HCl, 60; riboflavin, 25; pyridoxine HCl, 50; folic acid, 10; dlcapantothenic acid, 75; nicotinic acid, 5; biotin, 1; inositol, 5.

**Table 2:** Primer sequences for RT-qPCR in the hepatopancreas of red claw crayfish.

Gene	Primer Sequence (5′ → 3′)	Amplicon Size (bp)	Tm (°C)	Genbank	R^2^	Slope	Efficiency (%)
*β-actin* ^1^	F: ATCACTGCTCTGGCTCCTGCTACC	148	60	XM_053800818.1	0.996	−3.30	100.92
	R: CGGACTCGTCGTACTCCTCCTTGG						
*acc* ^2^	F: GTCAGGAAGTTTGGAGGCAA	136	60	XM_053793522.1	1.000	−3.50	93.07
	R: TGAAATGAAAGGCACGGTCA						
*cpt1* ^3^	F: ACACCTGCCTATTGGTTGGG	142	60	XM_053797361.1	0.984	−3.40	96.84
	R: CTCAAGTCTTGGTGGGCTCC						
*adipor* ^4^	F: AACGACAGTGTACGCCTCTG	155	60	XM_053787771.1	0.991	−3.35	98.84
	R: TGAGGAAAGGCAGCGTGAAG						
*ampkα* ^5^	F: TTAAGTGGTGACCGAGGAGT	186	60	XM_053773531.1	0.998	−3.38	97.63
	R: AAACCTCGCTCATGATGTCC						
*ampkβ* ^6^	F: ATGATGTAGGCTCGCAGAAC	108	60	XM_053796745.1	0.993	−3.48	93.80
	R: GCGGCTTGTCCTTATTGTTG						
*ampkγ* ^7^	F: CTGACCCCTTCCTGGAAAAC	97	60	XM_053773266.1	0.998	−3.46	94.54
	R: AGGTCATAGGTGTGGTGGAA						
*pparγ* ^8^	F: CGGATGGTGGTGGAGGAGTAGG	81	60	XM_053785396.1	0.998	−3.45	94.92
	R: CCCAGGAGCCAACGACAACAC						
*srebp* ^9^	F: GTCTTCCTGGTGGGTCTCCTCTAC	85	60	XM_053788865.1	0.997	−3.25	103.09
	R: CGACTTCCGCCTCACTCTCAATG						
*akt* ^10^	F: GGAAGCAGCAGCAGCAGTGG	136	60	XM_053793380.1	0.998	−3.41	96.45
	R: TGGAAGTTCGGCGGTCAATCATC						
*mtor1* ^11^	F: GGAGGAGGAGGAAGAAGAGGAAGC	142	60	XM_053785700.1	0.986	−3.31	100.50
	R: GCACAACACCAGAGCCACACTC						
*mtor2* ^12^	F: GTAGCGGCACTGCGGGTTG	175	60	XM_053771218.1	0.985	−3.47	94.17
	R: TTGGTGGGAGGTGGCTGGTG						
*fas* ^13^	F: TTGACTTCAAAGGTCCCAGC	126	60	XM_053782425.1	0.969	−3.57	90.60
	R: GGTTAGTACCTCCCACCACT						

**Note:** ^1^ *β-actin*: Non-regulated reference gene. ^2^ *acc*: acetyl-CoA carboxylase. ^3^ *cpt1*: carnitine palmitoyl transferase 1. ^4^ *adipor*: Adiponectin receptor. ^5^ *ampkα*: Adenosine monophosphate-activated protein kinase alpha. ^6^ *ampkβ*: Adenosine monophosphate-activated protein kinase beta. ^7^ *ampkγ*: Adenosine monophosphate-activated protein kinase gamma. ^8^ *pparγ*: Peroxisome proliferator-activated receptor gamma. ^9^ *srebp*: Sterol regulatory element-binding protein. ^10^ *akt*: Protein kinase B. ^11^ *mtor1*: Rapamycin target protein 1. ^12^ *mtor2*: Rapamycin target protein 2. ^13^ *fas*: Fatty acid synthase.

**Table 3:** Effects of *Lactobacillus plantarum* supplementation on survival rate and final weight in red claw crayfish.

Index	Groups				F-Value	*p*-Value
	CK	LG	MG	HG		
Survival rate (%)	88.89 ± 0.56 ^c^	92.78 ± 0.56 ^b^	95.56 ± 0.56 ^a^	91.67 ± 0.96 ^b^	29.878	<0.001
Final weight (g)	7.66 ± 0.11 ^c^	8.54 ± 0.17 ^b^	9.50 ± 0.22 ^a^	8.28 ± 0.17 ^b^	58.947	<0.001

**Note:** All the data are presented as mean ± SD (*n* = 3). Different superscript letters in the same row indicate significant differences among the data (*p* < 0.05).

**Table 4:** Effects of *Lactobacillus plantarum* supplementation on muscle composition in red claw crayfish (%/dry matter).

Index	Groups				F-Value	*p*-Value
	CK	LG	MG	HG		
Crude protein	7.90 ± 0.20	7.92 ± 0.30	8.00 ± 1.33	7.95 ± 0.51	1.020	0.433
Crude lipid	2.79 ± 0.08	2.85 ± 0.01	2.88 ± 0.04	2.84 ± 0.02	1.826	0.221
Ash	6.38 ± 0.08	6.37 ± 0.02	6.38 ± 0.06	6.40 ± 0.02	0.118	0.947
Moisture	79.47 ± 1.25	79.57 ± 1.82	79.93 ± 1.15	79.97 ± 0.81	0.481	0.704

**Note:** All the data are presented as mean ± SD (*n* = 3).

**Table 5:** Effects of *Lactobacillus plantarum* supplementation on the muscle amino acid composition in red claw crayfish (g/100 g dry matter).

Index	Groups				F-Value	*p*-Value
	CK	LG	MG	HG		
Arginine	9.92 ± 0.07 ^d^	10.12 ± 0.07 ^c^	10.52 ± 0.07 ^a^	10.32 ± 0.07 ^b^	47.244	<0.001
Histidine	2.26 ± 0.05 ^c^	2.35 ± 0.03 ^b^	2.43 ± 0.03 ^a^	2.36 ± 0.04 ^ab^	10.608	0.004
Isoleucine	3.52 ± 0.04 ^b^	3.6 ± 0.03 ^ab^	3.75 ± 0.05 ^a^	3.78 ± 0.19 ^a^	4.339	0.043
Leucine	6.62 ± 0.07 ^c^	6.80 ± 0.03 ^b^	6.85 ± 0.05 ^ab^	6.88 ± 0.04 ^a^	19.253	0.001
Lysine	7.48 ± 0.06 ^b^	7.63 ± 0.05 ^a^	7.67 ± 0.08 ^a^	7.72 ± 0.02 ^a^	11.083	0.003
Methionine	2.06 ± 0.05 ^b^	2.15 ± 0.03 ^a^	2.20 ± 0.04 ^a^	2.16 ± 0.04 ^a^	7.441	0.011
Phenylalanine	3.32 ± 0.03 ^c^	3.38 ± 0.04 ^bc^	3.45 ± 0.03 ^a^	3.40 ± 0.03 ^ab^	8.255	0.008
Threonine	3.42 ± 0.04	3.44 ± 0.04	3.49 ± 0.06	3.50 ± 0.04	2.441	0.139
Valine	3.85 ± 0.05 ^b^	3.95 ± 0.03 ^a^	4.02 ± 0.04 ^a^	4.00 ± 0.04 ^a^	11.72	0.003
Total Essential Amino Acids	42.43 ± 0.34	41.15 ± 4.16	44.36 ± 0.18	44.03 ± 0.16	1.519	0.282
Alanine	4.42 ± 0.07 ^b^	4.51 ± 0.08 ^ab^	4.58 ± 0.08 ^a^	4.64 ± 0.05 ^a^	6.261	0.017
Aspartic acid	8.92 ± 0.07 ^c^	9.12 ± 0.07 ^b^	9.25 ± 0.05 ^a^	9.20 ± 0.03 ^ab^	22.479	<0.001
Glutamic acid	14.78 ± 0.08 ^c^	14.92 ± 0.07 ^b^	15.04 ± 0.04 ^a^	15.02 ± 0.03 ^a^	14.784	0.001
Glycine	3.80 ± 0.05	3.89 ± 0.03	3.82 ± 0.12	3.79 ± 0.13	0.700	0.578
Proline	3.55 ± 0.05 ^c^	3.68 ± 0.05 ^ab^	3.61 ± 0.04 ^bc^	3.70 ± 0.04 ^a^	7.523	0.001
Serine	3.53 ± 0.05 ^c^	3.60 ± 0.08 ^bc^	3.69 ± 0.08 ^ab^	3.73 ± 0.03 ^a^	6.359	0.016
Tyrosine	3.70 ± 0.05	3.72 ± 0.06	3.74 ± 0.05	3.78 ± 0.07	1.047	0.423
Total non-essential amino acids	42.69 ± 0.41 ^b^	43.43 ± 0.23 ^a^	43.73 ± 0.16 ^a^	43.86 ± 0.02 ^a^	13.689	0.002

**Note:** All the data are presented as mean ± SD (*n* = 3). Within the same row, different superscript letters indicate significant differences between the values (*p* < 0.05).

**Table 6:** Effects of *Lactobacillus plantarum* supplementation on muscle fatty acid composition in red claw crayfish (% of total fatty acids).

Index	Groups				F-Value	*p*-Value
	CK	LG	MG	HG		
C14:0	0.37 ± 0.02	0.38 ± 0.02	0.36 ± 0.02	0.35 ± 0.02	1.250	0.354
C16:0	1.54 ± 0.02	1.53 ± 0.02	1.52 ± 0.02	1.51 ± 0.02	1.250	0.354
C17:0	0.38 ± 0.01	0.38 ± 0.02	0.39 ± 0.02	0.38 ± 0.02	0.231	0.872
C18:0	8.73 ± 0.03 ^a^	8.71 ± 0.03 ^ab^	8.68 ± 0.03 ^ab^	8.65 ± 0.03 ^b^	4.083	0.05
∑SFAs ^1^	11.02 ± 0.08	10.99 ± 0.08	10.95 ± 0.09	10.89 ± 0.09	1.307	0.338
C16:1n-7	4.85 ± 0.05 ^d^	5.00 ± 0.05 ^c^	5.15 ± 0.05 ^b^	5.30 ± 0.05 ^a^	45.000	<0.001
C18:1n-9	25.5 ± 0.10 ^d^	25.9 ± 0.10 ^c^	26.4 ± 0.10 ^b^	26.9 ± 0.10 ^a^	110.750	<0.001
∑MUFAs ^2^	30.35 ± 0.15 ^d^	30.9 ± 0.15 ^c^	31.55 ± 0.15 ^b^	32.2 ± 0.15 ^a^	85.556	<0.001
C20:2n-9	0.86 ± 0.01 ^c^	0.87 ± 0.01 ^bc^	0.88 ± 0.01 ^ab^	0.89 ± 0.01 ^a^	5.000	0.031
C18:2n-6	14.35 ± 0.05 ^c^	14.50 ± 0.05 ^b^	14.65 ± 0.05 ^a^	14.7 ± 0.05 ^a^	30.000	<0.001
C18:3n-6	0.18 ± 0.01 ^c^	0.19 ± 0.01 ^bc^	0.2 ± 0.01 ^ab^	0.21 ± 0.01 ^a^	5.000	0.031
C20:3n-6	3.32 ± 0.02 ^c^	3.35 ± 0.02 ^bc^	3.38 ± 0.02 ^ab^	3.41 ± 0.02 ^a^	11.250	0.003
C20:4n-6 (ARA ^3^)	0.81 ± 0.01 ^d^	0.84 ± 0.01 ^c^	0.87 ± 0.01 ^b^	0.9 ± 0.01 ^a^	45.000	<0.001
C22:4n-6	1.07 ± 0.02 ^d^	1.12 ± 0.02 ^c^	1.17 ± 0.02 ^b^	1.22 ± 0.02 ^a^	31.250	<0.001
∑n-6PUFAs ^4^	20.59 ± 0.12 ^d^	20.87 ± 0.12 ^c^	21.15 ± 0.12 ^b^	21.43 ± 0.12 ^a^	27.222	<0.001
C18:3n-3	3.75 ± 0.05 ^d^	3.87 ± 0.05 ^c^	3.99 ± 0.05 ^b^	4.11 ± 0.05 ^a^	28.800	<0.001
C20:5n-3 (EPA ^5^)	13.15 ± 0.15 ^d^	13.65 ± 0.15 ^c^	14.35 ± 0.15 ^b^	15.15 ± 0.15 ^a^	100.778	<0.001
C22:5n-3	1.23 ± 0.03 ^d^	1.31 ± 0.03 ^c^	1.39 ± 0.03 ^b^	1.47 ± 0.03 ^a^	35.556	<0.001
C22:6n-3 (DHA ^6^)	3.65 ± 0.05 ^d^	3.77 ± 0.05 ^c^	3.89 ± 0.05 ^b^	4.01 ± 0.05 ^a^	28.800	<0.001
∑n-3 PUFAs ^7^	21.78 ± 0.28 ^d^	22.60 ± 0.28 ^c^	23.62 ± 0.28 ^b^	24.74 ± 0.28 ^a^	62.800	<0.001
∑PUFAs ^8^	42.37 ± 0.38 ^d^	43.47 ± 0.40 ^c^	44.77 ± 0.40 ^b^	46.17 ± 0.40 ^a^	52.774	<0.001
∑LC-PUFAs ^9^	21.18 ± 0.28 ^d^	21.91 ± 0.28 ^c^	22.73 ± 0.28 ^b^	23.57 ± 0.28 ^a^	40.756	<0.001
n-3/n-6	1.06 ± 0.01 ^d^	1.08 ± 0.01 ^c^	1.12 ± 0.01 ^b^	1.16 ± 0.01 ^a^	59.000	<0.001
DHA + EPA	16.80 ± 0.20 ^d^	17.42 ± 0.20 ^c^	18.24 ± 0.20 ^b^	19.16 ± 0.20 ^a^	78.588	<0.001

**Note:** All the data are presented as mean ± SD (*n* = 3). Data were subjected to one-way analysis of variance (ANOVA) to evaluate the effects of dietary treatments on muscle composition. Within the same row, different superscript letters indicate significant differences between the values (*p* < 0.05). ^1^ ∑SFAs: Saturated fatty acids; ^2^ ∑MUFAs: Monounsaturated fatty acids; ^3^ ARA: Arachidonic acid; ^4^ ∑n-6 PUFAs: n-6 Polyunsaturated fatty acids; ^5^ EPA: Eicosapentaenoic acid; ^6^ DHA: Docosahexaenoic acid; ^7^ ∑n-3 PUFAs: n-3 Polyunsaturated fatty acids; ^8^ ∑PUFAs: Polyunsaturated fatty acids; ^9^ ∑LC-PUFAs: Long-chain polyunsaturated fatty acids.

**Table 7:** Effects of *Lactobacillus plantarum* supplementation on biochemical parameters of hemolymph in red claw crayfish.

Index	Groups				F-Value	*p*-Value
	CK	LG	MG	HG		
AKP ^1^ (King unit/100 mL)	18.76 ± 0.24 ^c^	20.01 ± 0.56 ^b^	21.86 ± 0.15 ^a^	21.38 ± 0.15 ^a^	18.886	<0.001
CHE ^2^ (U/L)	172.36 ± 2.55	183.86 ± 13.28	186.98 ± 15.90	184.18 ± 2.49	0.380	0.770
GLU ^3^ (mmol/L)	4.55 ± 0.17 ^b^	4.70 ± 0.11 ^b^	5.89 ± 0.08 ^a^	4.78 ± 0.18 ^b^	19.022	<0.001
AST ^4^ (U/L)	2.51 ± 0.14 ^a^	1.80 ± 0.17 ^b^	1.07 ± 0.30 ^c^	1.75 ± 0.13 ^b^	8.853	0.006
ALT ^5^ (U/L)	1.65 ± 0.08 ^a^	0.93 ± 0.10 ^b^	0.78 ± 0.07 ^b^	1.01 ± 0.07 ^b^	22.491	<0.001
LDH ^6^ (U/L)	1476.92 ± 106.59 ^a^	1027.69 ± 54.70 ^b^	953.85 ± 81.41 ^b^	1015.38 ± 53.29 ^b^	9.765	0.005
TP ^7^ (g/L)	879.90 ± 13.13 ^c^	937.68 ± 24.07 ^b^	1084.77 ± 11.45 ^a^	984.96 ± 16.40 ^b^	26.092	<0.001
LZM ^8^ (U/mL)	212.12 ± 6.31 ^c^	258.59 ± 11.65 ^b^	310.10 ± 4.40 ^a^	258.59 ± 2.67 ^b^	31.722	<0.001
T-CHO ^9^ (mmol/L)	2.84 ± 0.39	2.82 ± 0.01	2.84 ± 0.22	2.84 ± 0.01	0.171	0.913
ALB ^10^ (g/L)	18.83 ± 0.11 ^b^	19.88 ± 0.38 ^b^	23.16 ± 20.43 ^a^	19.67 ± 0.32 ^b^	22.028	<0.001

**Notes:** All the data are presented as mean ± SD (*n* = 3). Data were subjected to one-way analysis of variance (ANOVA) to evaluate the effects of dietary treatments on muscle composition. Within the same row, different superscript letters indicate significant differences between the values (*p* < 0.05). ^1^ AKP: Alkaline phosphatase. ^2^ CHE: Cholinesterase. ^3^ GLU: Glucose. ^4^ AST: Aspartate aminotransferase. ^5^ ALT: Alanine aminotransferase. ^6^ LDH: Lactate dehydrogenase. ^7^ TP: Total protein. ^8^ LZM: Lysozyme. ^9^ T-CHO: Total cholesterol. ^10^ ALB: Albumin.

## Data Availability

The original contributions presented in the study are included in the article, and further inquiries can be directed to the corresponding authors.
